# Building on Success: Assessment Categories for Experimental Noncancer End Points

**DOI:** 10.1289/ehp.117-a112

**Published:** 2009-03

**Authors:** Bob Weinhold

Since 1978 the National Toxicology Program (NTP) has coordinated, developed, carried out, and informed the public about toxicology testing within the federal government. For chemical carcinogenesis bioassays, the NTP has long used a set of standardized criteria for classifying the relative strength of experimental outcomes. These criteria have enabled the NTP to assess the potential carcinogenicity of nearly 600 substances. Now the program is launching similar criteria for assessing other types of adverse effects, such as damage to the immune system, the reproductive system, and the developing organism.

The new criteria attempt to impose a sense of uniformity to different types of toxicology data. “It’s very difficult to develop criteria for noncancer end points,” says Nancy Kerkvliet, a professor of immunotoxicology at Oregon State University, a former member of the NTP Board of Scientific Counselors (BSC), and chair of the Working Group that reviewed the new immunotoxicity classification criteria. “There’s such a variety of end points. It’s difficult to rank them for importance.” The NTP, aware of this inherent difficulty, hopes the levels-of-evidence criteria will allow better transference to public health decisions.

Decades of cancer studies have provided a widely accepted template for assessing and evaluating the relative comparability and importance of various cancer study outcomes. First created in 1983, the NTP’s 5-tiered classification system describes the strength of evidence for conclusions for NTP chemical carcinogenesis studies. These classifications—which include 1) clear evidence, 2) some evidence, 3) equivocal evidence, 4) no evidence, and 5) inadequate study—are used to frame the conclusions per sex/species in the experiment cited in each NTP technical report. “These classifications have stood the test of time,” says Paul Foster, NTP discipline leader for reproductive and developmental toxicology.

The strongest findings, which can be considered “clear evidence” of carcinogenic activity, are demonstrated by studies that are interpreted as showing a dose-related increase of malignant neoplasms, a combination of malignant and benign neoplasms, or benign neoplasms if there is any indication that such tumors could progress to malignancy. To help NTP staff decide on borderline cases, there are 15 additional reference points, or factors to be considered in addressing various aspects, such as “presence or absence of dose relationships” and “statistical significance of the observed tumor increase.” Using these criteria allows NTP staff to better develop consistent conclusions for multiple studies of the same substance and for comparing different chemicals under these same criteria.

The NTP classification system is applied only to those studies conducted by the program. The 5 designations are intended to be solely a conclusion about whether a substance may potentially pose a carcinogenic hazard to humans. They are not intended to describe a carcinogenic risk to human health, but to be an integral part of the more formal risk assessment process that takes place at state and other federal agencies. This formal risk assessment requires additional evaluation of factors such as specific doses, routes, and likelihood of exposure as well as a whole host of other information including published bioassays and toxicology studies conducted by different organizations.

## Similar Classifications for Other Toxicologic Outcomes

The long-term successful use of the carcinogenicity classification system suggested to NTP leaders that they could develop similar classifications for other toxicologic outcomes for which there were adequate experimental and scientific data—in particular, toxicity to the immune and reproductive systems and the developing organism.

To start the process, Foster and Dori Germolec, NTP leader for immunotoxicology, consulted with internal toxicologists and external colleagues to devise wording for 5 categories of evidence. Next, the NTP BSC, which routinely provides scientific advice to the organization, formed two Working Groups comprising members from federal and state regulatory agencies, academia, corporations, and nongovernment organizations. “The composition of the two Working Groups was carefully developed to include individuals who are experts in the conduct of immunotoxicology or reproductive/developmental studies, and individuals who might be users of the NTP studies such as regulatory agencies, in order to get critical input on the potential utility and applicability of the proposed criteria,” says Mary Wolfe, director of the NTP Office of Liaison, Policy, and Review.

These Working Groups reviewed the draft NTP criteria, and individuals applied the criteria to sample data sets, then compared their results as a group. The reports and recommendations of the Working Groups were presented to the BSC in November 2008. After review of the criteria by the BSC, Foster presented the Working Group reports to the NTP Executive Committee, whose members are drawn from 8 federal agencies. The new NTP criteria will be presented at the Society of Toxicology meeting in Baltimore, Maryland, in March 2009.

## Putting the New Categories into Practice

The criteria may be applied to chemicals using findings from previous NTP studies as a practice exercise, Germolec says. However, the criteria will not be used to evaluate new NTP reports until late 2009 and early 2010, when results are expected for gum guggul (an oleoresin used as a dietary supplement), resveratrol (an antioxidant produced by grape plants), tetrachloroazobenzene (a pesticide by-product), and an unnamed endocrine-disrupting fungicide (because the chemical is undergoing blind tests, its name will be released only upon completion of the study).

Once added to the developmental, reproductive, and immune system toxicity reports published by the NTP, the criteria will give readers a more standardized vocabulary for understanding the NTP’s interpretation of study results and better ability to compare results between chemicals. Among those who use the NTP reports—and base decisions upon them—are regulators, policymakers, politicians, lawyers, and advocacy groups.

Some observers suggest the criteria may require further adjustments. David Wegman, a professor of epidemiology at the University of Massachusetts Lowell and a BSC member, questions the “some evidence” category covering the broad swath between “clear” and “equivocal” evidence. “It’s an awful big range to cover with the word ‘some,’” Wegman says. He suggests possibly dividing that category into two better-defined categories.

Wegman also says that many details in the exact language used in the criteria still need to be refined. He admits that even he, as a long-term expert in the field of epidemiology, didn’t understand some of the terms as they were used by experts in other fields during the Working Group and BSC meetings. Because NTP reports are used by regulators and scientists in a variety of fields, he says, it is important that all users be able to understand the language describing the conclusions. And he believes others will face greater challenges. “The level of [broad-based] health literacy in our population is extremely poor,” he says. “So the language used has to be easily understood.”

NTP staff have refined the terms and language used to address concerns such as Wegman’s. In the preamble to each of the criteria documents, which will be available on the NTP website (http://ntp.niehs.nih.gov/) in early March, the NTP defines each of the 5 criteria as they apply to the individual areas of immunotoxicity, reproductive toxicity, and developmental toxicity. The documents include examples of the types of findings that support the assignment of one criterion over another and define key terms such as “dose-related.” The documents also point out the differences between hazard assessment—which is the intended use of these classifications—and the broader, more comprehensive process of formal risk assessment.

On a related note, there “was a significant amount of debate about the use of dose information [in applying the criteria],” Foster says. Dose information typically falls into the domain of risk assessment, but there’s a majority agreement so far that some kind of exposure information is needed if the criteria are to be applied realistically. “Hazard information without some kind of dose information is scary,” says Kim Boekelheide, a professor of medical science at Brown University and a member of the Working Group that reviewed the reproductive and developmental toxicity criteria. Each NTP technical report will include information about the lowest dose at which an adverse effect was observed as well as complete details used to reach conclusions.

The NTP is looking into how to develop criteria for other organ and system toxicities, such as neurologic, gastroenterologic, cardio vascular, and pulmonary end points. For now, the NTP and many of those who use its reports look forward to introducing and using their classifications for immunotoxicity, reproductive toxicity, and developmental toxicity.

“Right now we don’t have anything in the way of categories of evidence,” says Edward Carney, technical leader for developmental, reproductive, and general toxicology at The Dow Chemical Company and chair of the reproductive and developmental toxicity Working Group. “In the end this system will be better than what we have now.” James Donald, chief of the Reproductive Toxicology and Epidemiology Section at the California EPA Office of Environmental Health Hazard Assessment and a member of the reproductive and developmental toxicity Working Group, agrees: “I think for NTP’s purposes it’s going to be very useful.”

## Figures and Tables

**Figure:**
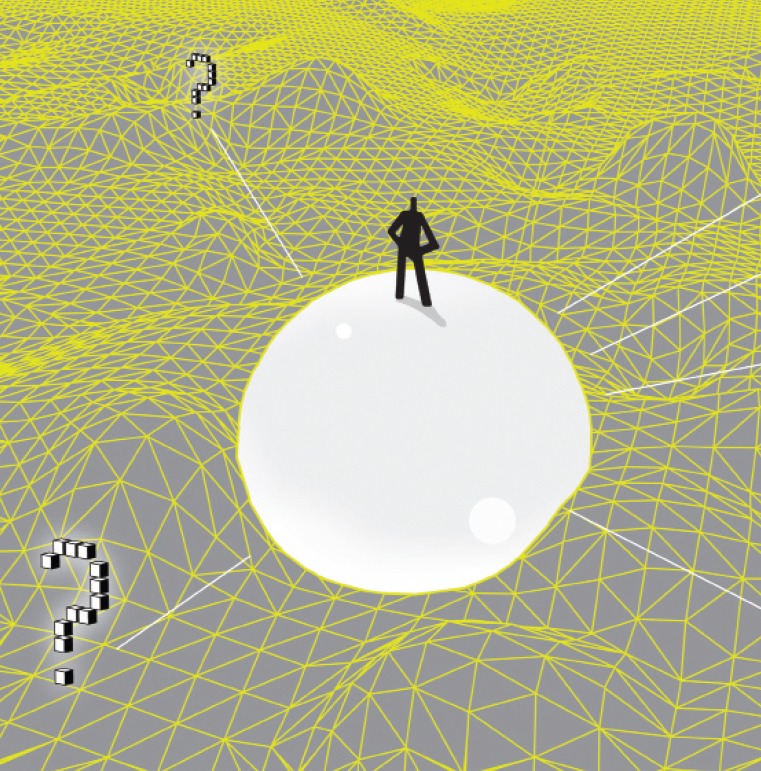


**Figure:**
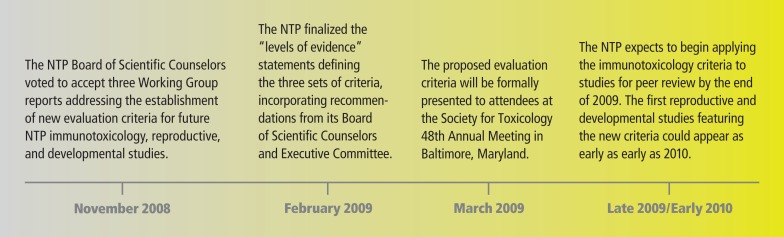
Timeline for Noncancer Evaluation Criteria

